# Challenges in the diagnosis and management of acromegaly: a focus on comorbidities

**DOI:** 10.1007/s11102-016-0725-2

**Published:** 2016-06-08

**Authors:** Alin Abreu, Alejandro Pinzón Tovar, Rafael Castellanos, Alex Valenzuela, Claudia Milena Gómez Giraldo, Alejandro Castellanos Pinedo, Doly Pantoja Guerrero, Carlos Alfonso Builes Barrera, Humberto Ignacio Franco, Antônio Ribeiro-Oliveira, Lucio Vilar, Raquel S. Jallad, Felipe Gaia Duarte, Mônica Gadelha, Cesar Luiz Boguszewski, Julio Abucham, Luciana A. Naves, Nina Rosa C. Musolino, Maria Estela Justamante de Faria, Ciliana Rossato, Marcello D. Bronstein

**Affiliations:** Endocrinology Unit, Centro Médico Imbanaco Cali, Cali, Colombia; Internal Medicine Department, Hospital of Neiva, University Surcolombiana, Neiva, Colombia; Internal Medicine Department, University Industrial of Santander, Bucaramanga, Colombia; Department of Internal Medicine, Endocrinology Fundación Cardio-Infantil, Instituto de Cardiología, Universidad del Rosario, Bogotá, Colombia; Endocrinology Unit, Hospital Universitario San Ignacio, Bogotá, Colombia; Internal Medicine Department, Hospital San Jerónimo de Montería, Montería, Córdoba, Colombia; Endocrinology Unit, Hospital Universitario Departamental de Nariño, CENTRO de Endocrinologia CENDOO, Universidad Nacional de Colombia, Pasto, Colombia; Endocrinology Department, Hospital Universitario San Vicente Fundación, Universidad de Antioquia, Medellín, Colombia; Endocrinology Faculty, Universidad Caldas, Manizales, Colombia; Federal University of Minas Gerais, Belo Horizonte, Brazil; Endocrinology and Chair, Division of Endocrinology, Hospital das Clínicas, Pernambuco Federal University Medical School, Recife, Brazil; Neuroendocrine Unit, Division of Endocrinology and Metabolism, Hospital das Clínicas, University of São Paulo Medical School, Av. Dr. Eneas de Carvalho, 255, 7ºandar, sala 7037, São Carlos, SP CEP 05403-000 Brazil; Endocrine Section, Medical School and Hospital Universitário Clementino Fraga Filho, Universidade Federal do Rio de Janeiro, Rio de Janeiro, Brazil; Department of Internal Medicine, Endocrine Division (SEMPR), Federal University of Paraná, Curitiba, Brazil; Neuroendocrine Unit, Escola Paulista de Medicina, Universidade Federal de São Paulo, São Paulo, SP Brazil; Department of Endocrinology, Faculty of Medicine, University of Brasilia, Brasília, Brazil; Neuroendocrine Unit, Division of Neurosurgery, Hospital das Clínicas, University of Sao Paulo School of Medicine, São Paulo, SP Brazil; Department of Odontology, Central Unit, Hospital das Clínicas, University of Sao Paulo School of Medicine, São Paulo, SP Brazil

**Keywords:** Acromegaly diagnosis, Acromegaly comorbidities, Latin America

## Abstract

**Introduction:**

Acromegaly is a rare, insidious disease resulting from the overproduction of growth hormone (GH) and insulin-like growth factor 1 (IGF-1), and is associated with a range of comorbidities. The extent of associated complications and mortality risk is related to length of exposure to the excess GH and IGF-1, thus early diagnosis and treatment is imperative. Unfortunately, acromegaly is often diagnosed late, when patients already have a wide range of comorbidities. The presence of comorbid conditions contributes significantly to patient morbidity/mortality and impaired quality of life.

**Methods:**

We conducted a retrospective literature review for information relating to the diagnosis of acromegaly, and its associated comorbidities using PubMed. The main aim of this review is to highlight the issues of comorbidities in acromegaly, and to reinforce the importance of early diagnosis and treatment.

**Findings and conclusions:**

Successful management of acromegaly goes beyond treating the disease itself, since many patients are diagnosed late in disease evolution, they present with a range of comorbid conditions, such as cardiovascular disease, diabetes, hypertension, and sleep apnea. It is important that patients are screened carefully at diagnosis (and thereafter), for common associated complications, and that biochemical control does not become the only treatment goal. Mortality and morbidities in acromegaly can be reduced successfully if patients are treated using a multimodal approach with comprehensive comorbidity management.

## Introduction

Acromegaly is a rare, insidious disease commonly caused by a pituitary adenoma, which overproduces growth hormone (GH), and results in increased levels of insulin-like growth factor 1 (IGF-1) [1–3]. Some studies estimate that it affects 86–240 per million of the population [4], though others have estimated its prevalence at 40–70 per million [5, 6]. Despite being a rare disease, its related comorbidities and the life-long management required mean that acromegaly can be a large burden [5]. Unfortunately, the delay between the initial appearance of symptoms and diagnosis can be very long, often in the range of 7–10 years [7–9]. If left untreated, the prolonged exposure to GH and IGF-1 is associated with worsening of comorbidities [7, 10, 11], and poorer quality of life [9, 12] and increased mortality risk [7, 10, 11]. Furthermore, a delay in diagnosis has been associated with psychosocial impairment, including depression, body image distortion, and social withdrawal [13, 14]. As such, early diagnosis and treatment is extremely important to allow for earlier initiation of appropriate treatment, which in turn would lead to more successful disease management and ultimately better outcomes.

### Diagnosis

There have been several key advances over the last two decades in this disease, including the development of assays with increased sensitivity for GH and IGF-1 [15, 16], widespread use of magnetic resonance imaging (MRI) for pituitary examination [17], automated software to evaluate the physical changes associated with the disease [18], and several novel therapeutic approaches [19]. Despite this, the diagnosis of these patients does not seem to have changed in over 25 years, nor have the signs and symptoms associated with the disease [9]. Most patients still have marked manifestations of acromegaly at diagnosis, suggesting that acromegaly remains clinically under-recognized.

Although acromegaly manifests with distinct physical characteristics, diagnosis of the disease in its early stages can be difficult due to its insidious nature, meaning that neither the patient, their families nor their physicians may notice these changes. The recognition that acromegaly can be accompanied by apparently normal GH concentrations and dynamics, mild or absent clinical features and pituitary tumors that are too small to be detected by MRI, must contribute to this difficulty, and indicates the importance of IGF-1 measurement in diagnosis [20]. A number of pre-existing illnesses, including catabolic states, hepatic or renal failure, malnutrition and diabetes mellitus may lower the IGF-I level and result in false-negative values. The presence of certain key comorbid conditions (e.g., orofacial changes) can serve as an important diagnostic pointer to the presence of acromegaly, and the presence of multiple commodities (diabetes, arthralgias, cardiomyopathy, etc., summarized in Table 1) may also serve to raise suspicion to its presence. There is a clear role to be played for specialists from other disciplines (e.g., dentists), as well as primary care physicians, in the early identification of patients with acromegaly through better awareness of common comorbidities. For example, studies have shown that around 40 % of patients are diagnosed by internists or family physicians, and also by ophthalmologists (if they have changes in vision), dentists (orofacial changes, separation of teeth, and malocclusion), gynecologists (menstrual irregularities and infertility), rheumatologists (joint problems), or pulmonologist (sleep apnea) [21, 22]. Many studies have provided diagnostic algorithms to help in the identification and confirmation of acromegaly [21, 23, 24], and one recent study, which has yet to be validated, has provided a scoring system to aid in the early recognition of signs and symptoms [25]. This scoring system, termed ‘ACROSCORE’, is intended to be used by general practitioners and nonendocrinology specialists to help in diagnosis by scoring symptoms, and depending on the final score, the patient can then be refereed for a more detailed investigation by endocrinologist. [25].

**Table 1:** Clinical features of acromegaly

Mass effects of tumor	Headache, visual impairment, hyperprolactinemia, pituitary stalk section, hypopituitarism, hypothyroidism, hypogonadism, hypocortisolism
Systemic effects of GH/IGF-1 excess	Soft tissue and skin changes, acral enlargement, increased skin thickness and soft tissue hypertrophy, increased sweating, skin tags and acanthosis nigricans
Cardiovascular features	Hypertrophy, congestive heart failure, coronary disease, arrhythmias, hypertension, cardiomyopathy
Metabolic features	Impaired glucose metabolism, diabetes, insulin resistance
Respiratory features	Macroglossia, upper airway obstruction, sleep apnea, ventilator dysfunction
Bone and joint features	Increased articular cartilage thickness, arthropathy/osteoarthritis, carpal tunnel syndrome, vertebral fractures
Other endocrine consequences	Goiter, hypercalciuria, menstrual abnormalities

Adapted from Cordero and Barkan [21] and Madeira et al. [66]

### Comorbidities associated with acromegaly and their management

A better awareness and approach, and in turn control, of acromegalic comorbidities (Table 1) may help improve mortality. Due to the delayed diagnosis in many patients there are often a number of comorbidities already present at diagnosis which can act as important indicators of the disease. In one study of 324 patients, the comorbidities present at diagnosis were recorded in two groups of patients, one treated between 1981 and 1984 (early) and the other treated between 1995 and 2006 (late) [9]. The most commonly present comorbidities at diagnosis in this study were hypertension, carpal tunnel syndrome, osteoarthritis, diabetes mellitus and sleep apnea. The study also shows that presenting comorbidities have not changed between 1981 and 2006 (Table 2). While early diagnosis and therapy are obviously very important, identification and treatment of disease comorbidities should be considered as equally important [20]. Improving treatment and control, of acromegalic comorbidities has the potential to normalize mortality in acromegaly patients similar to the general public [5]. Studies have shown that with disease cure or control, it is possible to reverse some, but not all, of the associated comorbidities. Ben-Shlomo et al. summarises the outcome of different comorbidities from several studies, and shows that a number of comorbidities can improve with disease control, including joint articular cartilage thickness, vertebral fractures, left ventricular function, exercise capacity and endurance, lipid profile, and obstructive apnea events [5]. However, many patients never achieve disease control [5].

**Table 2:** Comorbidities present at diagnosis in two groups of patients: one treated between 1981 and 1984 (early) and the other treated between 1995 and 2006 (late)

Comorbidity	Early group (%)	Late group (%)
Hypertension	37	36
Carpal tunnel syndrome	24	24
Osteoarthritis	25	23
Diabetes mellitus	18	15
Sleep apnea	13	29
Goiter	15	12
Malignancy	7.4	9.2
Kidney stones	8.0	8.0
Colon polyps	10	23
Skin tags	19	46

Adapted from Reid et al. [9]

### Cardiovascular complications

One of the most prevalent comorbidities in acromegaly patients is related to the cardiovascular system, including cardiomyopathy, atherosclerosis, and hypertension. If heart disease is present at diagnosis there is a mortality rate of 100 % within 15 years [10]. In one study, the 1- and 5-yearr mortality rates for patients with heart failure were 25 and 37.5 %, respectively [26]. Acromegaly may be associated with a specific cardiomyopathy, characterized by left ventricular hypertrophy, arrhythmias, decreased diastolic filling, and reduced left ventricular ejection fraction [10]. It is also associated with a high prevalence of hypertension. In a meta-analysis, mean prevalence of hypertension was 35 % (ranging from 18 to 60 %) in 18 series with 2562 patients [27]. Thus an early diagnosis and aggressive treatment of high blood pressure is imperative [10, 28]. If symptoms are persistent following biochemical control of acromegaly, standard treatment with antihypertensives, including thiazide-type diuretic, calcium channel blockers angiotensin converting enzyme, or angiotensin receptor blocker [29, 30]. There are conflicting data on the prevalence of atherosclerosis in acromegaly, with an increased incidence of carotid intima-media thickness being demonstrated by some authors, but not others [31, 32]. One study showed that the risk of coronary artery disease was low, and remained stable following successful treatment [33]. An integrated evaluation of the Framingham score and coronary artery calcium has shown that 40 % of acromegalic patients were at risk for coronary atherosclerosis and that coronary calcifications were evident in about half of them [34].

Somatostatin analogues, also known as somatostatin receptor ligands (SRLs) are associated with significant reductions in heart rate, left ventricular mass index, interventricular septum thickness, and left ventricular posterior wall thickness. They are also associated with improved exercise tolerance, and trends toward beneficial effects for left ventricular end-diastolic dimension and ejection fraction. However, the effects were not significant for blood pressure, left ventricular, end-systolic dimension, or fractional shortening [35]. Interestingly, SRL treatment may result in improvement of cardiovascular effects regardless of biochemical control, for example, one study has shown that acromegalic cardiomyopathy was improved in patients who did not achieve biochemical control [36]. Long-term treatment with pegvisomant, on the other hand, induced a slight reduction of carotid arteries wall thickness and a significant improvement of brachial arteries vascular function in patients with acromegaly resistant to somatostatin analogues [37]. Cardiovascular function has also been reported to improve following successful transsphenoidal surgery [38], however surgery is contraindicated in patients with severe cardiomyopathy. Since cardiovascular complications, such as hypertension and heart disease negatively determine life expectancy in acromegaly, adequate control of these aspects is highly relevant to reduce the morbidity and mortality associated with this disease.

### Pulmonary/respiratory comorbidities

Acromegaly patients are prone to respiratory problems, with mortality due to respiratory disease reaching 25 % of cases [10]. Impaired respiratory function originates from the multiple anatomical changes associated with the disease e.g., airway anatomy, bones, muscle structure of the chest, and lung elasticity [10]. Patients with acromegaly develop barrel chest due to changes in vertebral and costal morphology, and upper airway obstruction is a result of macroglossia, prognathism, thick lips, and hypertrophy of the laryngeal mucosa and cartilage, which can result in sleep apnea and excessive snoring. Hypoventilation and hypoxemia may also develop from central respiratory depression and kyphoscoliosis [39].

Diagnosis of pulmonary complications can be carried out using a chest x-ray, spirometry, and polysomnography. Evaluations for sleep apnea in particular should be considered, with prevalence ranging from 27 % in older studies to more than 80 % in more recent ones [40]. Interestingly, there may be a relationship between sleep apnea and other acromegaly comorbidities, such as insulin resistance and cardiovascular problems [40–42]. Pegvisomant has been reported to cure obstructive sleep apnea in some patients and somatostatin analogues are reported to improve sleep apnea, and reduce the apnea-hypoxia index by 50 % [43–45]. However, in prospective studies, sleep apnea persisted in more than 40 % of patients cured of acromegaly. Therefore, patients should be reassessed after efficacious acromegaly treatment to determine whether continued sleep apnea treatment is necessary [44]. If sleep apnea persists, standard guidelines for therapy should be followed, such as noninvasive ventilation and consultation with a maxillofacial surgeons is advised and if necessary elective surgery should be undertaken [46]. Acromegaly has also been linked to increased pulmonary infections, therefore patients should be given appropriate vaccinations against influenza and pneumococcal pneumonia [29].

### Malignancies

Studies on different types of cancer suggest that high ‘normal levels’ of IGF-I (that is, IGF-1 levels at the highest terciles) are related to an elevated cancer risk. Patients with acromegaly appear to have a higher prevalence of colon cancer [47]. There are a number of studies looking at the relationship between acromegaly and colon cancer, for which there is an increased odds ratio (2.04–4.351) [47, 48], although mortality from colon cancer does not appear to be increased [47]. In the colon, GH and IGF-1 excess leads to increased epithelial cell proliferation and decreased apoptosis rate [49]; normal and tumoral colorectal cells express large amounts of IGF-1 receptor [50, 51], and epithelial colorectal cells express the GH receptor [52]. Screening colonoscopy has been proposed by some groups to be carried out at diagnosis in adults, by the age of 40 or 50 by others [53] and patients should subsequently be checked as follows: (1) if colonoscopy is negative, and IGF-1 levels are normal, patients should be screened similar to the general population, (2) if colonoscopy is negative and IGF-1 levels remain elevated, more frequent screening (every 5–10 years) is recommended, and 3) if colonoscopy shows abnormalities, the follow-up should be repeated after 3–5 years [46, 54]. Repeated colonoscopic screening of patients with acromegaly has demonstrated a high prevalence of new adenomatous and hyperplastic colonic polyps, dependent on both the occurrence of previous polyps and elevated IGF-1 levels [55]. This illustrates the importance of close follow-up with these patients. Therapeutic management options for colon cancer include surgery, chemotherapy and radiotherapy, and do not differ from patients without acromegaly, however this cancer can largely be prevented by early screening and removal of adenomatous polyps [56].

In addition to colon cancer, there also appears to be a relationship between acromegaly and thyroid cancer, with reports of an increased prevalence of thyroid nodules and thyroid cancer in these patients [57, 58]. However, because the overall rate of benign and malignant tumors appears to be similar to the general population, patients should undergo standard periodical screening for thyroid function and morphology [10]. Although the presence of other cancers has been observed (e.g., breast, prostate, hematologic and cervical cancers), the screening in these cases should follow the same recommendation as in the normal population [59].

### Orofacial changes

Craniofacial development depends on genetic and ethnic characteristics, as well as on normal hormone secretion [60]. Normal development is characterized by the absence of asymmetries, normal maxilla-mandibular ratio, and dental contact in normal occlusion. In acromegaly there are soft tissue alterations (increased volume of tongue, uvula, lips and nose), and increased mandibular condyle-occlusion derangements [61, 62]. This can lead to functional disturbances, such as in chewing, swallowing, and speech. As the diagnosis of acromegaly is often delayed, orofacial changes such as prognathism, facial asymmetry, and dental diastemas may compromise the patient´s quality of life both functionally and socially. Since the dentist may be the first healthcare professional to examine the patient, they should be made aware of the disease through awareness programs. Unfortunately, unlike agromegalic changes to soft tissue, associated bone enlargement is not reversible with successful treatment. If any corrective surgical procedures are to be performed, this should only be carried out after normalization of GH and IGF-1 levels [29].

### Orthopedic and rheumatologic comorbidities

Arthropathy in acromegaly is common; one Brazilian study reported as many as 56 % of patients with arthropathy [63], and some studies reporting prevalence as high as 70 %. In many acromegaly patients, the earliest manifestations of arthropathy are articular manifestations [63–65]. Arthropathy and arthralgia may be reversible in the early stages of the disease, and adequate treatment reduces the risk of development, although it can progress even in the presence of normal levels of GH and IGF-I.

Bone disease is also highly prevalent, especially vertebral fractures with one study reporting a prevalence of around 10 % in acromegaly patients [66], and another reporting more than 40 % prevalence [64]. There appear to be specific risk factors for joint disease (female sex, age, obesity, duration of active acromegaly), and for arthropathy and vertebral fractures (age, hypogonadism, presence of active acromegaly) [63–65]. Early diagnosis of acromegaly can reduce the risk of joint disease and vertebral fractures, thereby improving quality of life. Adequate treatment of acromegaly reduces the risk of vertebral fractures [67], and treatment of hypogonadism improves bone mass and reduces the risk of fractures. In addition, weight loss improves joint pain in weight-bearing joints (knees and hip) [63, 64, 66]. In one study looking at the prevalence of osteoarticular changes in these patients, the authors suggest that it is important to be able to recognize and to include vertebral fracture assessments using, for example, lateral conventional radiographs of the spine in screening patients with acromegaly, both at diagnosis and during follow-up [68]. Many orthopedic/rheumatologic comorbidities persist despite biochemical control, for example, established degenerative arthritis may be irreversible, and painful arthropathy and joint complaints are a major contributor to a perceived reduced quality of life despite long-term biochemical remission [29]. Treatments for this type of persistent comorbidity should include physical therapy, anti-inflammatories and analgesic medications, with the consideration of joint replacement surgery [29]. Patients should also be assessed for osteoporosis risk factors, including vitamin D deficiency and inadequate calcium uptake [46].

### Metabolic disorders

Acromegaly is frequently associated with metabolic problems, such as diabetes and impaired glucose tolerance. Diabetes occurs more frequently in patients with acromegaly than the general population, and is an important predictive factor for increased mortality [7, 69]. In a recent study the prevalence of diabetes mellitus and early carbohydrate metabolic disorders considerably exceeds that of the general population [69]. A further study screening of 2270 individuals in Brazil with diabetes mellitus or glucose intolerance to evaluate the frequency of acromegaly in these adults, estimated the prevalence to be 480 cases per million adults [70]. Treatment of diabetes should be the same as for patients without acromegaly. Lowering GH levels improves glycemic control whatever mode of treatment is used [46]. However, modifications of glucose homeostasis induced by somatostatin analogues may have an overall minor clinical impact in acromegaly despite their significant improvement of GH and IGF-1 control [71]. In patients where somatostatin worsens glucose control by inhibiting insulin secretion, alternative therapies such as pegvisomant should be considered [29]. In studies with pegvisomant, glucose levels are reduced and insulin sensitivity is improved [72]. Furthermore, successful surgical treatment of acromegaly may improve or reverse the abnormalities in the metabolism of glucose, lipids and lipoproteins [38, 73, 74]. A recently-approved SRL, pasireotide, which binds to multiple somatostatin receptors, unlike first generation SRLs, appears to have superior efficacy compared to octreotide (a first generation SRL), and a similar tolerability profile with the exception of raised hyperglycemia [75, 76]. Although this may be more efficacious than other treatments in the same class, and therefore an attractive choice, the increased risk of hyperglycaemia should be considered and proactively managed if chosen.

### Endocrine disorders

Hormonal comorbidities are common in acromegalic patients, including hyperprolactinemia and hypopituitarism. Hyperprolactinemia develops in approximately 30 % of patients due to pituitary stalk compression or mixed tumor secretion of GH and PRL, and can occur with or without galactorrhea [24]. Hypopituitarism is usually considered to be due to compression and destruction of the normal pituitary gland and/or stalk by the expanding mass; particularly a macroadenoma, and occurs in approximately 40 % of patients [24]. It can also result from radiotherapy used to treat acromegaly, even after many years after the therapeutic procedure, requiring lifelong monitoring of pituitary function in these patients [77]. Patients who receive radiotherapy need lifelong monitoring of pituitary function as new deficits can occur up to 15 years or more after irradiation. Hypopituitarism may also be increased after surgical treatment for acromegaly [78], which can in turn influence cardiovascular risk [79], which is already increased in acromegalic patients.

### Neurologic disorders

Some studies have reported increased neurological conditions in acromegalic patients, including intracranial aneurysm, herniation of cerebellar tonsils, hearing loss, visual impairment, headache, and carpal tunnel syndrome. This may be a result of the growing pituitary adenoma and brain volume affecting local structures. One recent study found that 17 % of patients with acromegaly also had intracranial aneurisms, and that the presence of aneurisms correlated with initial serum values of GH [80]. The authors suggested that a neuroradiological evaluation of the intracranial circulation be done in acromegalic patients. In another study, the same group also found an increased prevalence of herniated cerebellar tonsils in these patients (15 %) compared to controls (7 %), and increased headache and visual problems [81]. The authors suggested that resolution of excess GH could improve on these findings. Hearing loss is also quite common in patients with acromegaly, with a prevalence ranging from 35 to 43 % [82–84], however the literature suggests there may be no improvement of hearing loss with hormone control [82].

Another common neurologic disorder that is commonly associated with acromegaly is carpal tunnel syndrome, and has varying reports of prevalence ranging from 18 to 84 % [85, 86]; it seems that this complication could be improved with GH normalization. However, if symptoms persist after biochemical control, patients may require surgical release [29]. Lastly, the prevalence of headache in acromegaly is reported in 37 to 87 % of patients, and can be a significant contributor to reduced quality of life [87]. The pathophysiology of pituitary tumor-associated headaches is not fully understood, and the resolution of headache after surgery or GH control is not always achieved [88]. However, large-scale placebo-controlled trials have shown that short-acting octreotide can reduce headache in 75–80 % of patients with acromegaly [89], and the use of slow-release monthly octreotide has been shown to reduce this symptom after the sixth injection [90]. Prospective trials of lanreotide therapy have also shown a significant reduction in headache in addition to the other symptoms of acromegaly [91]. Some of these treatments, however, can have a deleterious effect on patients, for example dependency, and possibly rebound headaches [92].

### Psychiatric disorders

Patients with acromegaly often have an impaired quality of life due to the multiple comorbidities associated with the disease [12], and it is recommended that a quality of life questionnaire be used routinely during diagnosis, treatment, and monitoring of the disease [93]. These patients are also reported to have associated psychiatric disorders, which contribute to their quality of life, such as increased anxiety and decreased self-esteem [94], depression [14], and cognitive impairment [93, 95]. Many of these disorders are thought to occur more frequently in acromegaly than other somatic disorders [96]. There are a number of other psychiatric disorders associated with the different stages of the disease (Summarized in Table 3). This warrants an interdisciplinary approach, which includes psychological evaluation and assistance, in the management of the disease.

**Table 3:** Symptoms in patients suffering from acromegaly before, during, and after diagnosis and treatment

Before diagnosis	After diagnosis/before surgery	After surgery
Increased irritability	Relief	PTSD^a^
Increased anxiety	Impatience	Sadness
Emotional liability	Psychological strain	Anger
Feeling uncertain	Guilt	Fatigue
Sadness	Social withdrawal	Sleep disorders
Sleep disorders	Body image distortion	Body image distortion
Difficulty concentrating	Fear (death, surgery, family welfare)	Competence disorders
High stress levels	Anger	Social withdrawal
Lower self-esteem	Helplessness	Limited interest in daily activities
Loss of control	Sadness	Impaired short-term memory
Sense of competence loss	Sleep disorders	Denial
	Restlessness	Avoidance of medical staff

Adapted from Furman and Ezzat [98] and Szczesniak et al. [14]

^a^Post-traumatic stress disorder

Studies have shown that both surgery and medical treatment of acromegaly can have a positive effect on physiological parameters [97], however in one study of patients with acromegaly who had biochemical remission, there was a higher prevalence of psychopathologic conditions and maladaptive personality traits compared with matched control subjects and patients with clinically nonfunctioning pituitary adenomas. These data raise the possibility of an irreversible effect of previous GH hypersecretion on mood and behavior [29].

## Conclusions

New opportunities exist for early identification and monitoring of acromegaly comorbidities, and efforts should be made towards continuous awareness of this disease among primary care physicians (including those in training), specialists, dentists, and other health professionals. The presence of comorbid conditions contributes significantly to patient morbidity/mortality and impaired quality of life, and appropriate management of these comorbid conditions has the potential to improve long-term outcomes. Hormonal control of acromegaly (e.g., with surgery and/or somatostatin analogues) may contribute to the management of some comorbid conditions. However, specific therapies/treatments may be also be required to control comorbid conditions (e.g., antihyperglycemic drugs, antihypertensives, various surgical interventions, psychological therapy). Thus, better awareness and a more aggressive approach to treat acromegaly comorbidities may contribute to improving quality of life and decreasing disease mortality.
